# Supplementary material for: Ancestral glycoprotein hormone-receptor pathway controls growth in C. elegans
Source: Front Endocrinol (Lausanne). 2023 Jun 20;14:1200407. doi: 10.3389/fendo.2023.1200407 (PMC10319355; doi:10.3389/fendo.2023.1200407)
Supplement: Supplementary file 1 [file DataSheet_1.pdf]

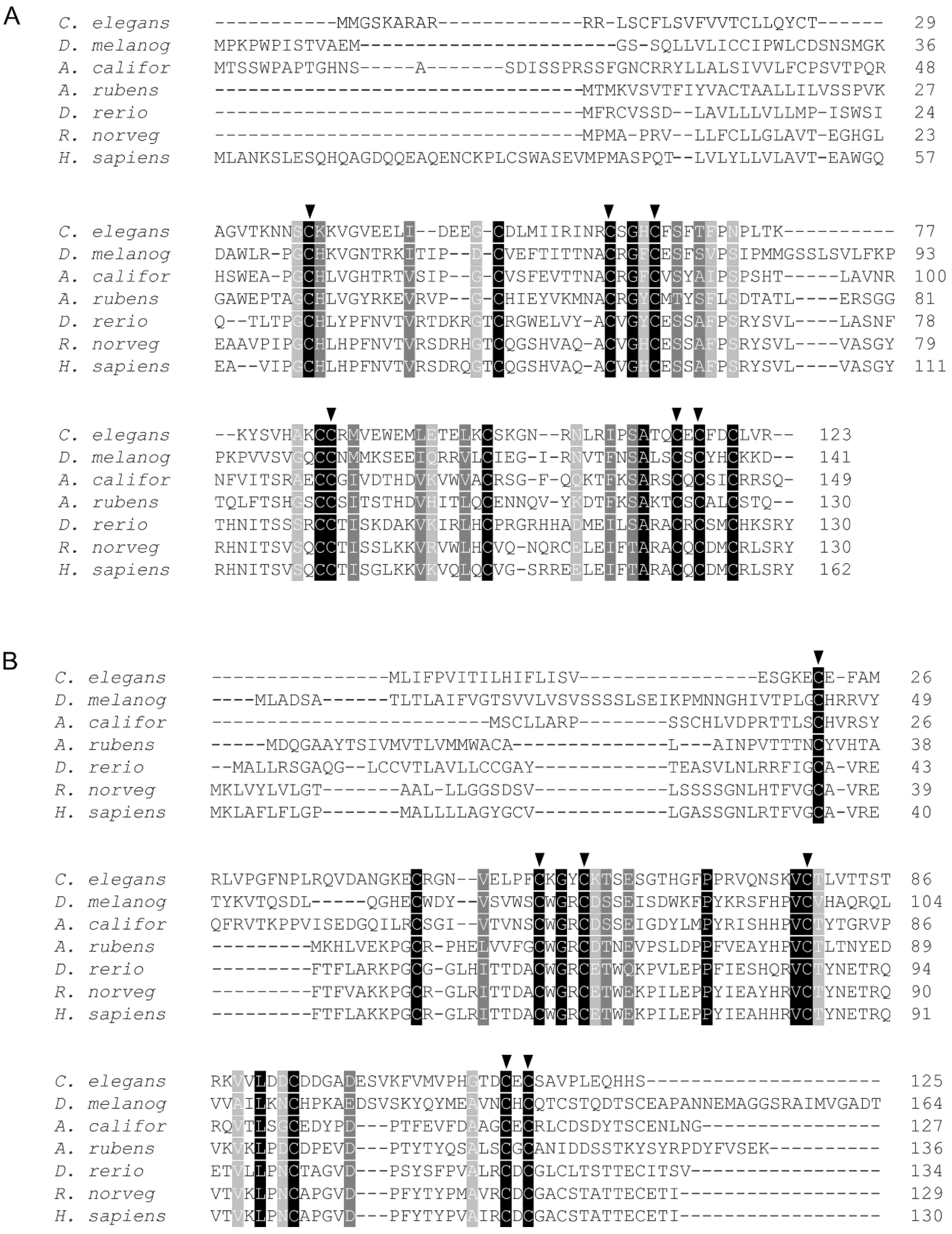

Figure S1. *C. elegans* GPLA-1 and GPLB-1 show sequence similarity with thyrostimulin GPA2 and GPB5. Protein sequence alignment of *C. elegans* GPLA-1 (A) and GPLB-1 (B) with GPA2 and GPB5 orthologs, respectively, in representative protostomian and deuterostomian species, including *Homo sapiens*, *Rattus norvegicus*, *Danio rerio*, *Asterias rubens*, *Aplysia californica*, and *Drosophila melanogaster* (Table S3). Six cysteine residues forming the typical cysteine knot structure of glycoprotein hormone subunits are highly conserved and indicated with black arrow heads. Black shading depicts full amino acid conservation, dark grey indicates groups of amino acids with strong similar properties, light grey depicts weakly similar amino acids.

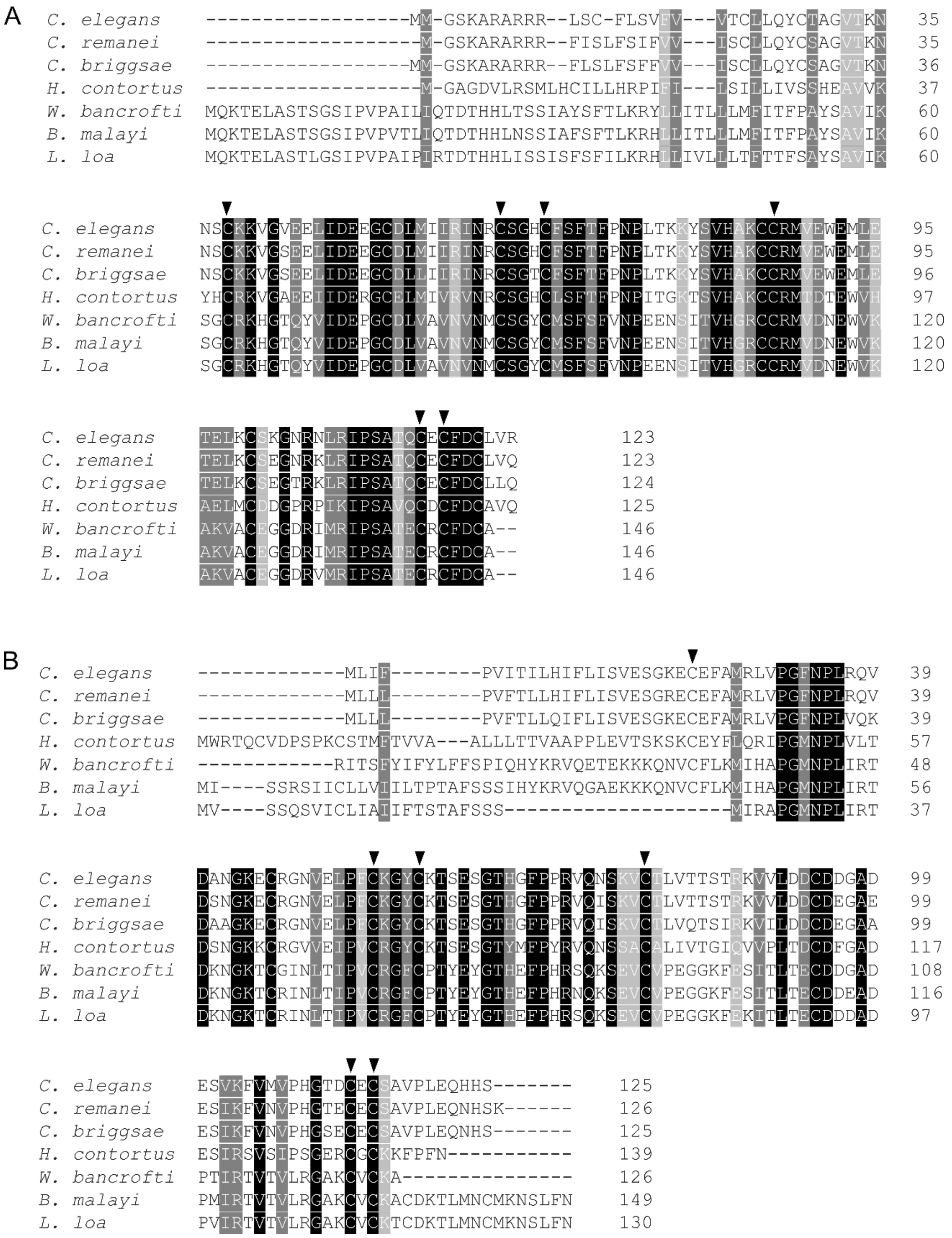

Figure S2. Conservation of thyrostimulin GPA2 and GPB5 subunits in nematodes. Protein sequence alignment of GPLA-1/GPA2 (A) and GPLB-1/GPB5 (B) sequences in nematode species, including *Caenorhabditis elegans, Caenorhabditis remanei, Caenorhabditis briggsae, Haemonchus contortus, Wuchereria bancrofti, Brugia malayi,* and *Loa loa* (Table S3). Symbols and shading as in figure S1.

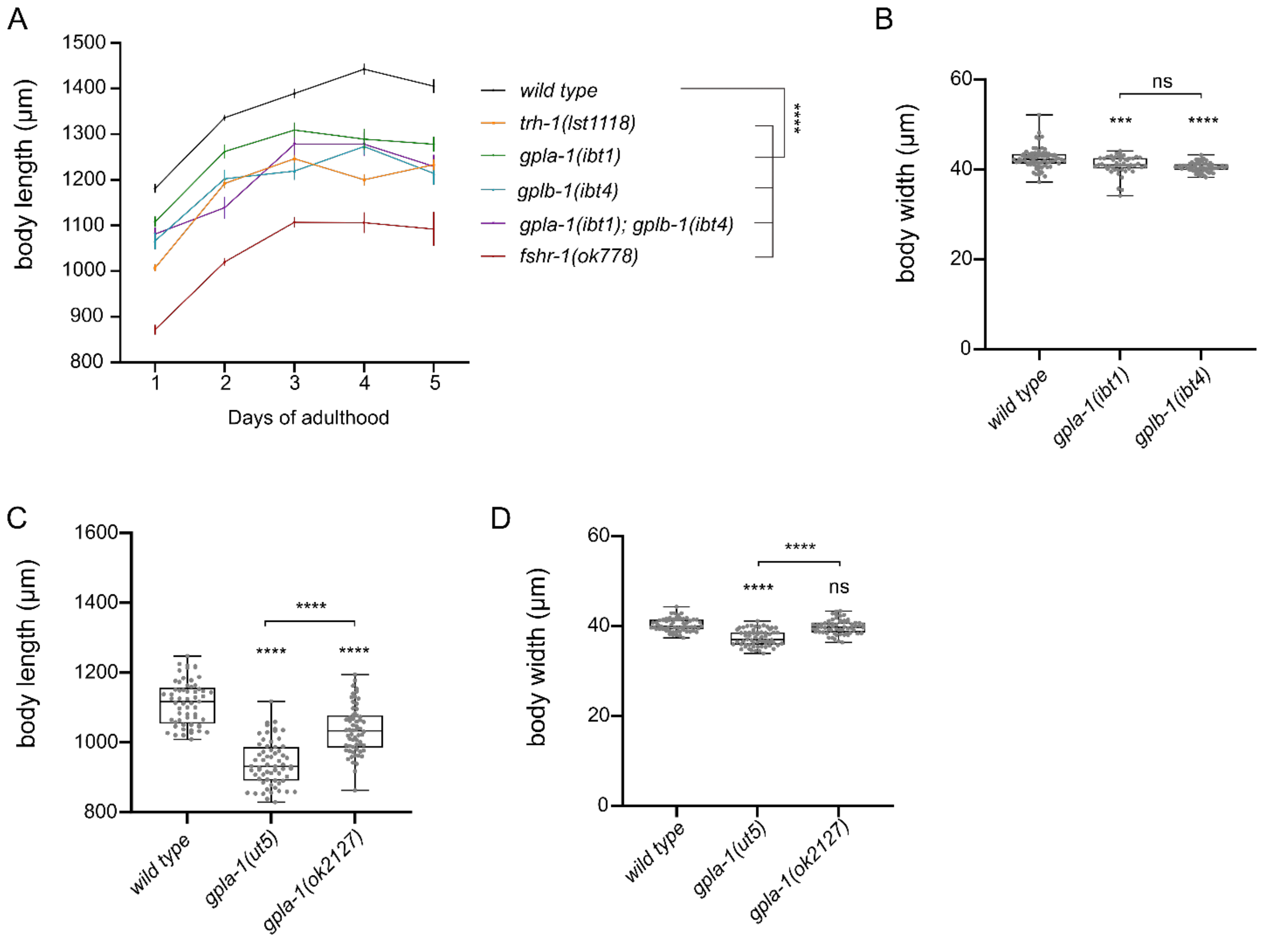

Figure S3. Mutants lacking thyrostimulin-like signaling show body size defects throughout adulthood. (A) Null mutants of *gpla-1*, *gplb-1* and *fshr-1* have a significantly shorter body length than wild-type animals throughout adult life, from day 1 to day 5 of adulthood. Mutants of the TRH-like neuropeptide *precursor trh-1* also have a shorter body length[1] and were used as positive control. Data is plotted as the mean body length ± SEM. For each genotype, around 20 animals were tested on each day of adulthood in at least 2 assays. (B) Comparison of body width of *gpla-1* and *gplb-1* mutants. (C-D) Comparison of body length (C) and width (D) of different *gpla-1* mutant alleles. Boxplots indicate 25th (lower boundary), 50th (central line), and 75th (upper boundary) percentiles. Whiskers show minimum and maximum values. For (B)-(D), each genotype was tested in 3 assays with 20-30 animals per trial. Data were analyzed by one-way ANOVA with Tukey's multiple comparisons test. ***P < 0.001; ****P < 0.0001; ns, not significant.

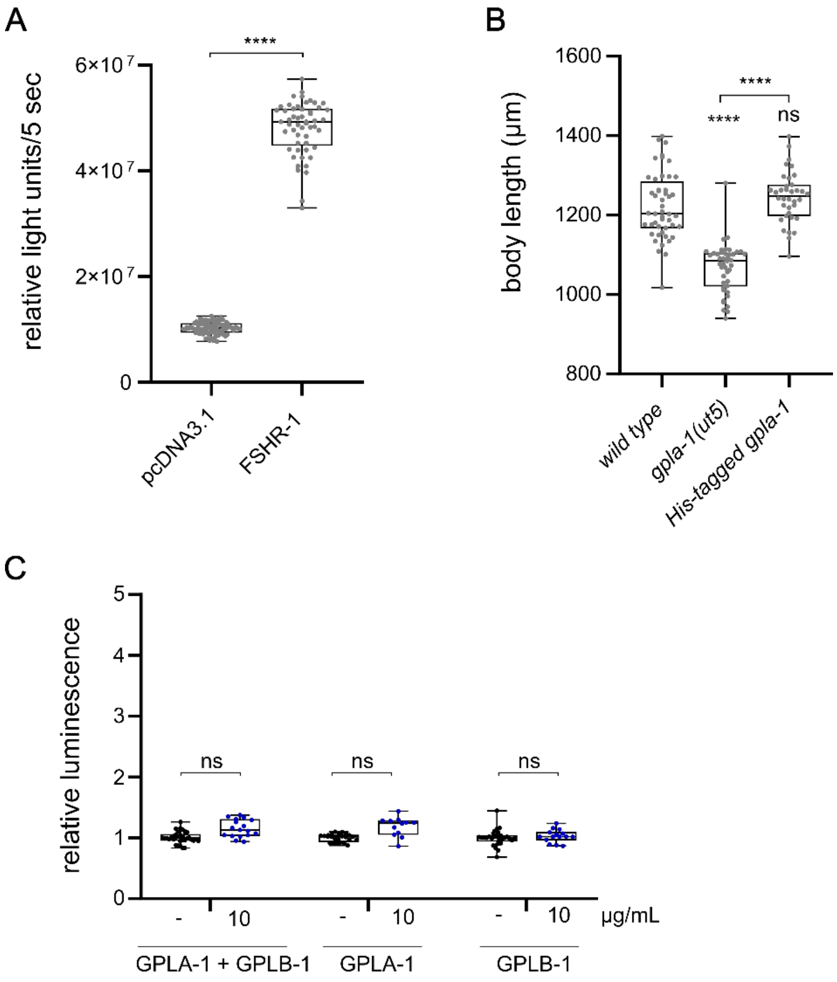

Figure S4. FSHR-1 shows basal activity in a cAMP-based receptor activation assay *in vitro*. (A) HEK cells expressing FSHR-1 show significantly higher cAMP-induced luminescence than cells transfected with an empty pcDNA3.1 vector control. Unpaired t test, n = 3 assays. (B) Transgenic *C. elegans* expressing N-terminal His-tagged GPLA-1 do not differ from wild-type animals in body length. Boxplots indicate 25th (lower boundary), 50th (central line), and 75th (upper boundary) percentiles. Whiskers show minimum and maximum values. Each genotype was tested in 2 assays with 20-30 animals per trial. One-way ANOVA with Tukey's multiple comparisons test. (C) Recombinant GPLA-1 and GPLB-1 proteins do not induce a significant increase in cAMP-mediated luminescence in cells transfected with an empty pcDNA3.1 vector control. Data are shown as relative luminescence after normalization to the ligand-free control. Two-way ANOVA with Dunnett's multiple comparisons test, ligand free controls, n ≥ 4 assays. ****P < 0.0001; ns, not significant.

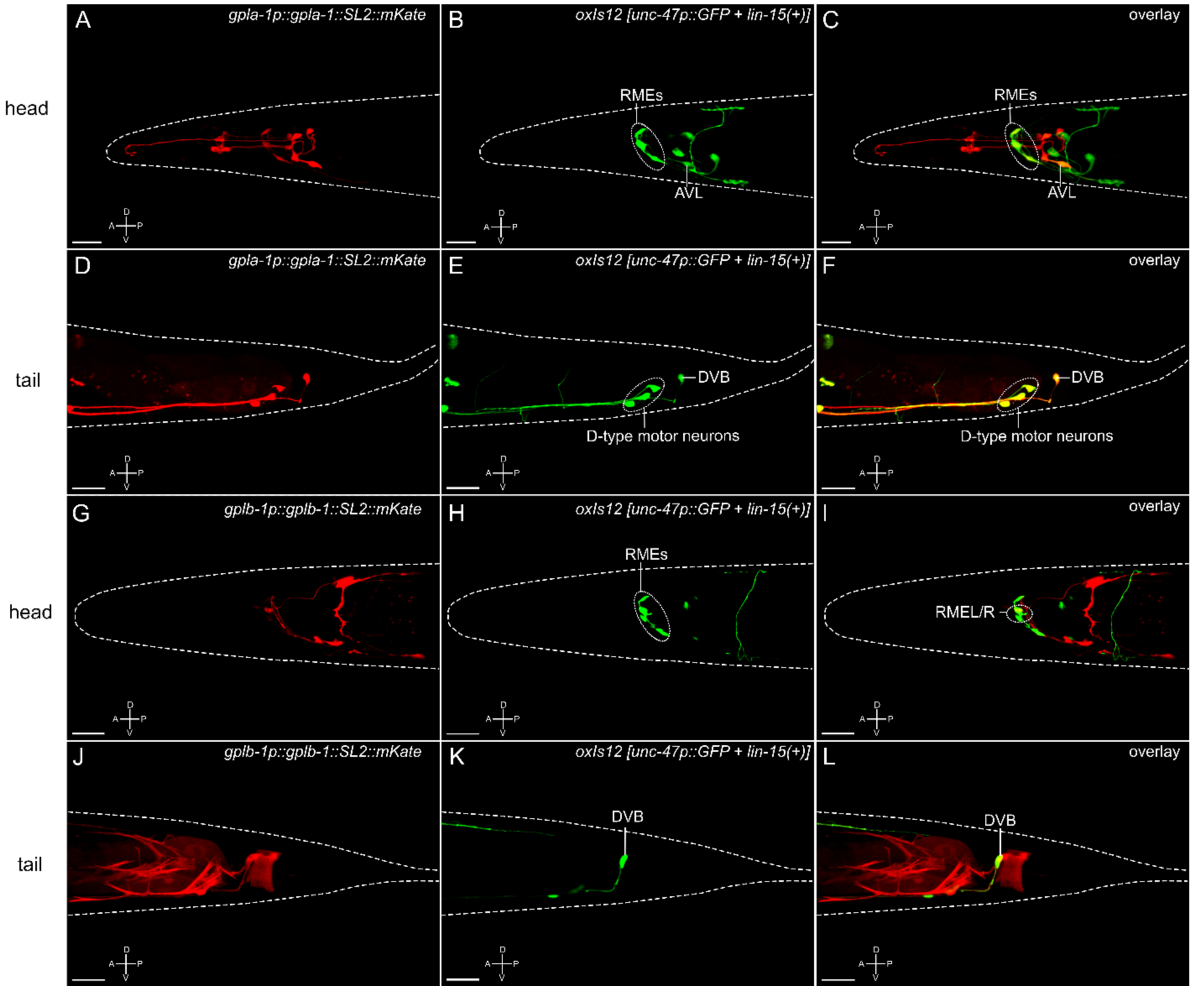

Figure S5. Identification of *gpla-1* and *gplb-1* expression sites in *C. elegans*. Labeled confocal Z-stack projections of *gpla-1* and *gplb-1* fluorescent reporter transgenes. Overlap with the GABAergic reporter strain EG1285 (*oxIs12 [unc-47p::GFP + lin-15(+)]*), marking GABAergic neurons, shows co-localization in RME and DVB neurons for *gpla-1* and *gplb-1*, and additionally in AVL and D-type motor neurons for *gpla-1*. A; anterior, P; posterior, D; dorsal, V; ventral orientation. Scale bars represent 20 µm.

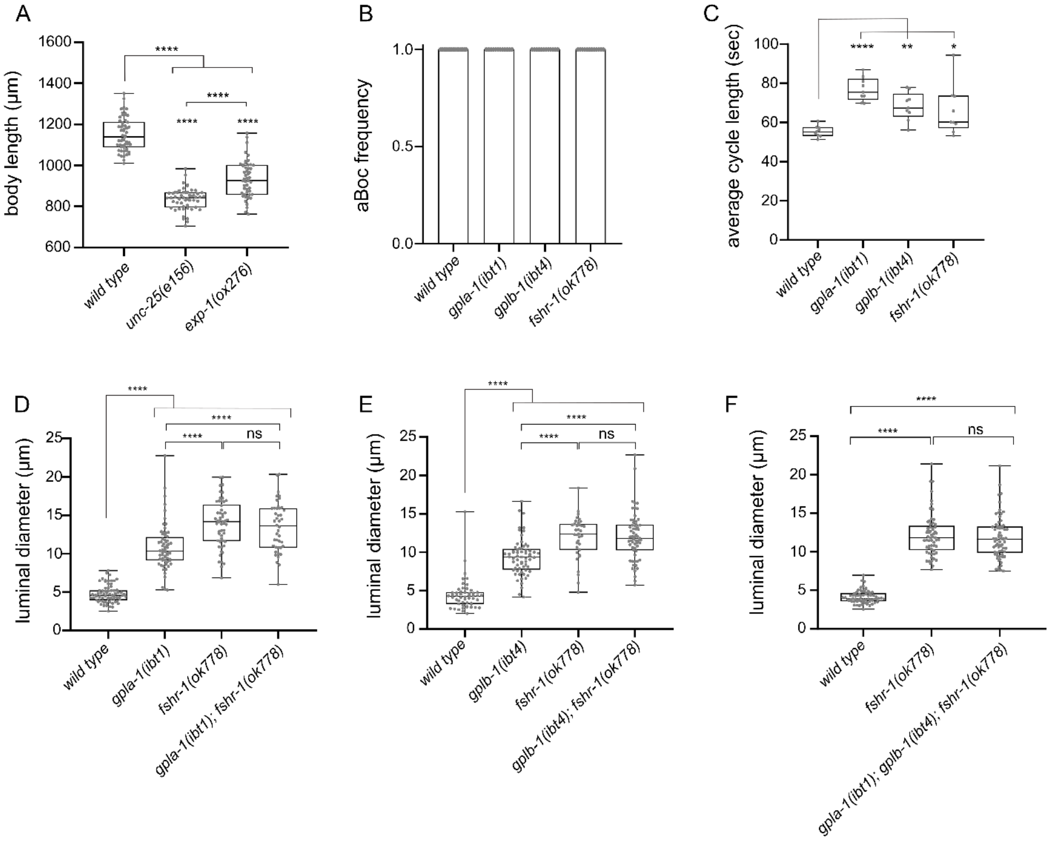

Figure S6. Mutants impaired in thyrostimulin-like signaling show defects in the defecation motor program and intestinal lumen size. (A) GABAergic transmission mutants *unc-25(e156)* and *exp-1(ox276)* that are defective in the defecation motor program[2–4] also show defects in body length. Each genotype was tested in 3 assays with at least 15 animals per trial. (B) The aBoc frequency *of gpla-1*, *gplb-1*, and *fshr-1* mutants does not differ from wild-type animals. Bar graphs depict the mean aBoc frequency ± SEM. One-way ANOVA with Dunnett's multiple comparisons test. (C) *gpla-1*, *gplb-1*, and *fshr-1* mutants show a significantly increased defecation cycle length compared to wild type. One-way ANOVA with Dunnett's multiple comparisons test. (D-E) Luminal width of double mutants lacking *fshr-1* and *gpla-1* (D) or *gplb-1* (E) does not significantly differ from the luminal width of single *fshr-1* mutants. One-way ANOVA with Tukey's multiple comparisons test. (F) The intestinal lumen of mutants lacking *gpla-1*, *gplb-1* and *fshr-1* is not significantly wider than that of single *fshr-1* mutants. One-way ANOVA with Tukey's multiple comparisons test. For (A) and (C)-(F), boxplots indicate 25th (lower boundary), 50th (central line), and 75th (upper boundary) percentiles. Whiskers show minimum and maximum values. For (B)-(C), each genotype was tested in 3 assays with 3 animals per trial. For (D)-(F), each genotype was tested in at least 2 assays with 20-30 animals per trial. *P < 0.05; **P < 0.01; ***P < 0.001; ****P < 0.0001; ns, not significant.

Table S1: Overview of *C. elegans* strains used in this study.

Strain name	Genotype	Figures
**N2**	Wild-type Bristol strain	Fig. 1B-E, Fig. 2E-F, Fig. 4A-F, Fig. 5A-E, Fig. S3A-D, Fig. S4B, Fig. S6A-F
**LSC1118**	*trh-1(lst1118) IV*	Fig. 5A-E, Fig. S3A
**IBE1**	*fshr-1(ok778) V* (4x outcrossed to N2)	Fig. 2E-F, Fig 4A, B and F, Fig. S3A, Fig. S6B-F
**IBE7**	*gpla-1(ut5) V* (4x outcrossed to N2)	Fig. S3C-D, Fig. S4B
**IBE24**	*gpla-1(ok2127) V* (5x outcrossed to N2)	Fig. S3C-D
**IBE50**	*ibtEx11 [gpla-1p::gpla-1 cDNA::sl2::mKate 50 ng/µl; unc-122p::gfp 25 ng/µl]*	Fig. 1E
**IBE88**	*gpla-1(ibt1) V* (4x outcrossed to N2)	Fig. 1A, B and D, Fig. 2F, Fig. 4B, C and D, Fig. 5A and D, Fig. S3A-B, Fig. S6B-D
**IBE89**	*fshr-1 (ok778) V; ibtEx15 [fshr-1p::fshr-1 gDNA::sl2::mKate 10 ng/µl; unc-122p::gfp 25 ng/µl]*	Fig. 2E, Fig. 3E-F, Fig. 4F
**IBE137**	*gpla-1(ibt3* [His::GPLA-1]*)*	Fig. S4B
**IBE141**	*trh-1(lst1118) IV; gpla-1(ibt1) V*	Fig. 5A and D
**IBE149**	*gpla-1(ibt1) V; fshr-1(ok778) V*	Fig. 2F, Fig. S6D

**IBE208**	*gplb-1(ibt4) V* (4x outcrossed to N2)	Fig. 1A, C and D, Fig. 2F, Fig. 4B, C and E, Fig. 5A and E, Fig. S3A-B, Fig. S6B, C and E
**IBE223**	*fshr-1 (ok778) V; ibtEx34 [rab-3p::fshr-1 cDNA::sl2::mKate 20 ng/µl; unc-122p::gfp 25 ng/µl]*	Fig. 4A and F
**IBE225**	*fshr-1 (ok778) V; ibtEx35 [ges-1p::fshr-1 cDNA::sl2::GFP 30 ng/µl; myo-2p::mCherry 10 ng/µl]*	Fig. 4A and F
**IBE228**	*ibtEx30 [gpla-1p::gpla-1 cDNA::sl2::mKate 20 ng/µl; unc-122p::gfp 25 ng/µl]; lin-15B(n765) X; oxIs12 [unc-47p::GFP + lin-15(+)]*	Fig. 3A-B, Fig. S5A-F
**IBE229**	*ibtEx10 [gplb-1p::gplb-1 cDNA::sl2::mKate 50 ng/µl; unc-122p::gfp 25 ng/µl]; lin-15B(n765) X; oxIs12 [unc-47p::GFP + lin-15(+)]*	Fig. 3C-D, Fig. S5G-L
**IBE333**	*fshr-1 (ok778) V; ibtEx51 [mir-228p::fshr-1 cDNA::sl2::mKate 20 ng/µl; unc-122p::GFP 10 ng/µl]*	Fig. 4A and F
**IBE265**	*gpla-1(ibt1) V; gplb-1(ibt4) V*	Fig. 1D, Fig. 4C, Fig. S3A
**IBE280**	*gpla-1(ibt1) V; ibtEx43 [gpla-1p::gpla-1 gDNA::sl2::GFP 40 ng/µl; unc-122p::dsRed 10 ng/µl]*	Fig. 1B, Fig. 4D
**IBE411**	*trh-1(lst1118) IV; gplb-1(ibt4) V*	Fig. 5A and E

**IBE418**	*gplb-1(ibt4) V; ibtEx61 [gplb-1p::gplb-1 gDNA a isoform::sl2::mKate 40 ng/µl; unc-122p::gfp 10 ng/µl]*	Fig. 1C, Fig. 4E
**IBE419**	*ibtEx62 [gplb-1p::gplb-1 cDNA::sl2::mKate 30 ng/µl; unc-122p::gfp 10 ng/µl]*	Fig. 1E
**IBE420**	*gplb-1(ibt4) V; fshr-1(ok778) V*	Fig. 2F, Fig. S6E
**IBE421**	*gpla-1(ibt1) V; gplb-1(ibt4) V; fshr-1(ok778) V*	Fig. 2F, Fig. S6F
**IBE465**	*fshr-1(ok778) V; ibtEx67 [rgef-1p::fshr-1 cDNA::sl2::GFP 40 ng/µl; myo-2p::mCherry 10 ng/µl]*	Fig. 4A and F
**EG1285**	*lin-15B&lin-15A(n765) oxIs12 [unc-47p::GFP + lin-15(+)] X*	Fig. S5
**CB156**	*unc-25(e156) III*	Fig. S6A
**EG276**	*exp-1(ox276) II*	Fig. S6A

Table S2: CRISPR RNA sequences used to target *gpla-1* and *gplb-1* with the respective 3' PAM sequences and repair sequences.

Gene	Targeting RNA sequence	PAM	Repair template
gpla-1	CCTATACCTTCTCAAATGAT	GGG	ATTTTTAACTTTTCAGCCTATACCTTCTCAAATGAGCATC TAAAAATGCAAAAAATCAAAGCGAAAGAAG
gpla-1	CTATAATAATCTCGTTGATA	CGG	
gplb-1	TATATGAAGTATAGTGATCA	CGG	GACGTTAAGCTCTTTTGAAAAAAATGCTTATATTTTTGA ACAACATCATTCATAAATTATCATACATTCA
gplb-1	TATGAATGATGTTGTTCAAG	TGG	

Table S3: Accession numbers of the alpha and beta glycoprotein hormone subunits used in this study.

Species	Glycoprotein hormone subunit alpha	Glycoprotein hormone subunit beta
Homo sapiens	XP_011543076.1	NP_660154.3
Rattus norvegicus	NP_598303.1	AAR92145.1
Danio rerio	ABR68845.1	NP_001159810.1
Asterias rubens	ALJ99964.1	ALJ99965.1
Aplysia californica	NP_001191641.1	NP_001191597.1
Drosophila melanogaster	NP_001104054.2	NP_001104335.1
Caenorhabditis elegans	BAI40098.1	NP_001123020.1
Caenorhabditis remanei	XP_003114490.1	XP_003110430.1
Caenorhabditis briggsae	CAP39678.2	ULT86177.1
Haemonchus contortus	CDJ89730.1	CDJ82236.1
Wuchereria bancrofti	VDM20099.1	VDM14690.1
Brugia malayi	XP_001901802.1	CRZ25575.1
Loa loa	XP_020302363.1	XP_020301644.1

References

1. Van Sinay E, Mirabeau O, Depuydt G, et al. Evolutionarily conserved TRH neuropeptide pathway regulates growth in *Caenorhabditis elegans*. *Proc Natl Acad Sci U S A* 2017;114(20):E4065-E4074. doi:10.1073/pnas.1617392114

2. Beg AA, Jorgensen EM. EXP-1 is an excitatory GABA-gated cation channel. *Nature Neuroscience* 2003;6(11):1145-1152. doi:10.1038/nn1136

3. Jin Y, Jorgensen E, Hartwieg E, Horvitz HR. The *Caenorhabditis elegans* gene *unc-25* encodes glutamic acid decarboxylase and is required for synaptic transmission but not synaptic development. *J Neurosci* 1999;19(2):539-548. doi:10.1523/jneurosci.19-02-00539.1999

4. Mclntire SL, Jorgensen E, Kaplan J, Horvitz HR. The GABAergic nervous system of *Caenorhabditis elegans*. *Nature* 1993;364(6435):337-341. doi:10.1038/364337a0
